# Chronic psychological stress and its impact on the development of aggressive breast cancer

**DOI:** 10.1590/S1679-45082015AO3344

**Published:** 2015

**Authors:** Thayse Fachin Cormanique, Lirane Elize Defante Ferreto de Almeida, Cynthia Alba Rech, Daniel Rech, Ana Cristina da Silva do Amaral Herrera, Carolina Panis

**Affiliations:** 1 Universidade Estadual do Oeste do Paraná, Francisco Beltrão, PR, Brazil.; 2 Hospital do Câncer de Francisco Beltrão, Francisco Beltrão, PR, Brazil.; 3 Pontifícia Universidade Católica do Paraná, Londrina, PR, Brazil.

**Keywords:** Stress, psychological, Breast neoplasms, Overweight, Obesity, Monitoring, immunologic

## Abstract

**Objective:**

To investigate the clinicopathological findings of women diagnosed with breast cancer and study the impact of chronic psychological stress on the pathological characteristics of these tumors.

**Methods:**

We investigated a cohort composed of women diagnosed with breast cancer and divided into two groups. One group was categorized as presenting with chronic psychological stress (by using the Self-Reporting Questionnaire − SRQ-20). Another group of women with breast cancer, but with no previous history of chronic psychological stress, comprised the Control Group. Clinical and pathological data were assessed.

**Results:**

Women presenting with a history of chronic distress were significantly overweight when compared to the Control Group. Furthermore, it was observed that these stressed women also had a significant percentage of aggressive breast cancer subtype, the HER2 amplified tumor, which could be putatively associated with the loss of immunosurveillance.

**Conclusion:**

Our findings suggested an interaction among chronic psychological stress, overweight, and the development of more aggressive breast tumors.

## INTRODUCTION

Breast cancer is a multifactorial disease, consisting of a public health problem worldwide. Some factors that interact among themselves contribute to the high incidence of breast cancer, including family history, presence of high-susceptibility genes, excessive body weight, and chronic stress.^1-3^

In this context, chronic psychological stress is a common finding reported by cancer patients. Stressful life events are considered important components that can affect the emotional state of the individuals, and their association with loss of social support is even related to significantly shortened survival in breast cancer patients.^4^ There is a significant positive association between early life distress and breast cancer development.^5^ Furthermore, a systematic analysis of some studies published in the last 30 years, investigating the causal attributions in breast cancer patients, demonstrated that breast cancer patient survivors consistently associate their disease with emotional distress, among another factors.^6^

Concerning the biological impact of chronic distress, the sustained psychological stress alone can lead to weight gain through several biological mechanisms,^2^ which may potentially result in loss of immunosurveillance.^7^ Competent immune responses are the major defense against cancer; therefore, their impairment is strongly associated with the development of several types of cancer, including breast tumors that confer poor prognosis.^8^

Although the relation between cancer development and chronic stress has been described, little is known concerning the impact of chronic psychological stress in the phenotype of breast tumors. To clarify this question, we investigated if women presenting with chronic emotional distress could exhibit more aggressive phenotypes of breast cancer.

## OBJECTIVE

To investigate clinicopathological findings from women diagnosed with breast cancer, and study the impact of chronic psychological stress in the pathological characteristics of tumors.

## METHODS

### Study design

This study included women living in the State of Paraná, diagnosed with infiltrative carcinoma of the breast, in the period from August 2013 to July 2014. They had been previously scheduled to undergo chemotherapy on a set day of the week, the same period. The selection of patients was conducted at the *Centro de Oncologia de Francisco Beltrão* (Ceonc), in the city of Francisco Beltrão (PR), Brazil. The inclusion criteria adopted were women with infiltrative ductal carcinoma of breast, uni- or bilateral, diagnosed between August 2013 and July 2014, eligible according to the Self-Reporting Questionnaire (SRQ-20) as stress or non-stress cohorts. To determine the sample size, we applied the following statistical calculation, in which: N0 = size number, Z = confidence interval, P = probability, D = error margin, n = sample size, and N = population size:


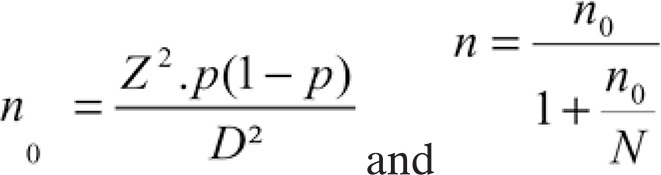


Considering that (1) our population was approximately 100 thousand inhabitants; (2) a p value of 0.05; (3) according to the* Instituto Nacional de Câncer *estimate, the incidence of breast cancer in this region was roughly 61 cases per 100 thousand women; (4) there were about 30 thousand women in the age range of risk for breast cancer in this area, thus, we have that the minimal sample size of about 18 patients was needed. Taking into account the period of the study and the fact that we worked with patients previously scheduled on a fixed day of the week to undergo chemotherapy, we decided to include 17 patients in each group. In this context, this study enrolled 34 women diagnosed with infiltrative ductal carcinoma of the breast. This study was previously approved by the Institutional Review Board, and all participants signed the Informed Consent Form. The research project was approved by the National Council of Scientific Research Ethics Committee, under number 497.050 and CAAE: 22027213.7.0000.0107. Clinical records were assessed and the data collected included age at diagnosis, weight, height, comorbidities, TNM classification, and chemotherapy regimen.

For determining the chronic psychological stress status of patients, we conducted an initial interview to verify the social support of the patient (family income, type of residence, level of education, life style, and social relationships), since we did not know if the patients were from different social conditions. Because all patients reported similar socioeconomic data, we continued the study by applying the SRQ-20 for psychiatric disorder screening.^9^ This interview was applied to patients that were in the hospital for routine chemotherapy treatment during the period of the study.

The women enrolled in this study were categorized into two groups: Control Group (n=17), with women diagnosed with breast cancer with no previous history of chronic psychological stress; Stress Group (n=17), with women diagnosed with breast cancer with a past history of chronic psychological stress.

### Molecular subtyping of breast tumors by immunohistochemistry

Formalin-fixed, paraffin-embedded samples from tumor biopsies were immunostained with primary antibodies for estrogen receptor (ER; anti-human estrogen receptor alpha, clone 1D5 at 1:600; Dako, Dinamarca), progesterone receptor (PR; anti-human progesterone, clone PGR 636 at 1:500; Dako, Dinamarca), and human epidermal growth receptor 2 (HER-2, anti-human HER2-pY-1248, clone PN2A at 1:500; Dako, Dinamarca), in association with a commercial immunohistochemistry kit. Samples were considered positive for ER/PR when at least 10% of the tumor cell nuclei were stained. HER2 was considered overexpressed when strong membrane staining (3+) was detected or when amplification of HER2 in samples with moderate (2+) membrane staining was observed in Fluorescent in Situ Hybridization (FISH) analysis. Samples were scored and categorized. They were considered HER2-positive when the HER2 IHC score was 3+, and HER-negative when the score was 1+ or zero. Samples with a 2+ score were analyzed by FISH to detect HER2 amplification (HER2 FISH pharmDx™; Dako, Dinamarca). Samples with a 2+ IHC score and an amplified result in FISH were considered HER2-positive, while samples with a 2+ IHC score and a non-amplified in FISH were considered HER2-negative.^10^

### Data analysis

For clinicopathological parameters, data were expressed as the mean ± standard error of the mean. All data were compared by using the non-parametric Mann-Whitney test. A p value <0.05 was considered significant. All statistical analyses were performed using the GraphPad Prism software version 5.0 (GraphPad Prism Software, San Diego, CA, USA).

## RESULTS

The mean age at diagnosis was 60.2 years. We found that 40% of patients were diagnosed as having breast cancer by routine mammograms, and 53% reported perception of nodules in the breasts by self-examination. The remaining 7% had no palpable nodules, but felt pain in breast and went to a doctor for this reason. Regarding the verification of chronic psychological stress (Table 1), it was observed that 79% reported a history of chronic psychological stress. The most common traumas related were death of family members, abandonment by a partner, employment loss, sexual abuse, and major depression. Moreover, 73% of patients associated the occurrence of chronic psychological stress with breast cancer development. A total of 47% were classified as potential carriers of psychic disorders by the SRQ-20. The patients who reported a positive history of psychological stress were categorized to form the Stress Group.

**Table 1:** Main parameters employed for chronic psychological stress characterization

Parameter	Mean percentage
Family relationships	
Good	85
Types of psychological distress reported	
Death of relatives	36
Loss of employment	9
Abandonment by partner	18
Sexual abuse	9
Depression	9
Other emotional distress	19
Received support during the treatment	
Yes	93
Presented difficulty for abandoning work	
Yes	57
Believes that chronic stress is associated as a cause of the disease	
Yes	73
Risk for developing mental disorders	
Yes	47

**Figure 1:**
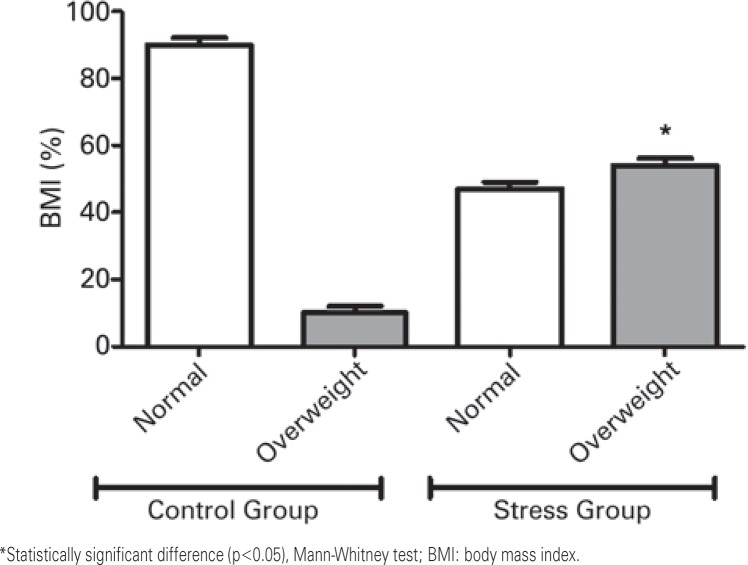
Analysis of body mass indexes. We used the International Classification of Body Mass Index to categorize the patients into normal weight (BMI up to 25kg/m2) or overweight (BMI above 25kg/m2)

**Figure 2:**
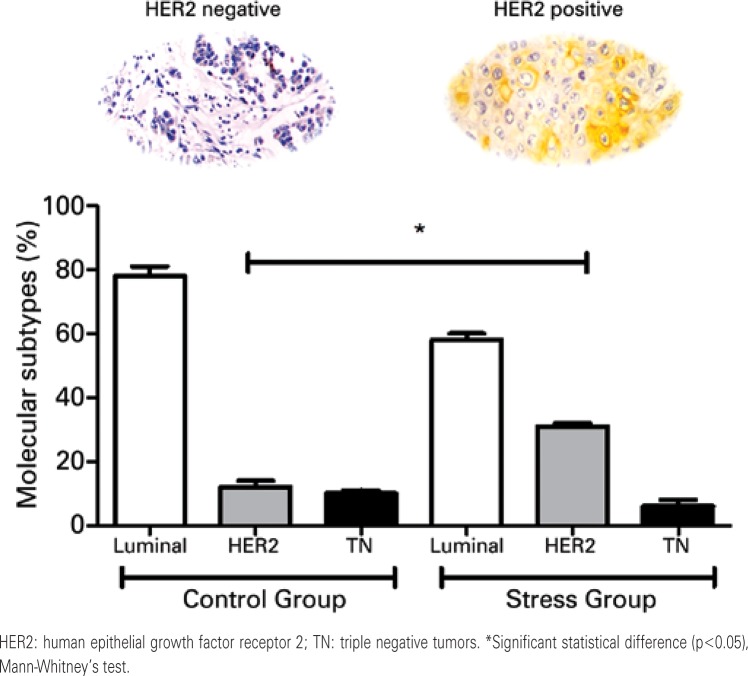
Molecular subtyping of tumors. The images represent the IHC labeling for HER2

Aiming to understand the clinicopathological impact of chronic psychological stress on breast cancer and clinical features, we compared the data from the Stress Group with a control cohort, composed of women diagnosed with breast cancer with no past history of chronic emotional stress. In this context, figure 1 shows that the Control Group comprised mainly women with breast cancer presenting with a normal body mass index (BMI) (90%). On the other hand, patients from the Stress Group exhibited a significant predominance of overweight women (54%; p=0.0041). The molecular subtyping of breast tumors (Figure 2) indicated the predominance of HER2 positive tumors in the Stress Group when compared to controls (31±1.41% *versus* 12±0.05%; p=0.0136).

## DISCUSSION

For the last years, breast cancer has been the leading malignant neoplasm in women worldwide. This fact deserves attention, since the list of risk factors related with this disease is growing. Our aim was to investigate women diagnosed with breast cancer who reported a past history of chronic psychological stress and its impact on clinicopathological aspects of breast cancer. To reach this goal we initially applied the SRQ-20 as a psychiatric screening tool to compose the stress cohort.

The SRQ-20 is an instrument developed by the World Health Organization (WHO) to screen psychiatric disorders in patients. It detects symptoms, such as anxiety, depression, and psychosomatic complaints with adequate accuracy.^11^ The SRQ-20 was previously applied and validated in the Brazilian population,^9^ including breast cancer patients.^12^ Our data demonstrated that about 47% of patients seen during the period of the SRQ-20 application were categorized as presenting with psychiatric distress and included in the Stress Group.

We further selected another cohort of patients who had a similar clinical history (age at diagnosis, family history, general health status, and family income), but with no prior report of chronic psychological stress to compose the Control Group. We employed this approach aiming to compare the impact of chronic emotional distress on clinical and pathological characteristics of breast tumors, in both groups. Our data indicated that a significant portion of the women enrolled in the Stress Group were overweight when compared to those of the Control Group. Both psychological stress and overweight are important risk factors for breast cancer development.^1,2^ Under chronic stress conditions, there is a systemic rise in cortisol levels, which increases appetite and favors the storage of lipids in adipose tissue.^2^ This set of biological events that contributes to weight gain is made up of relevant risk factors for breast cancer in the modern society,^8^ and may partly help to understand the results found in the present study. Overweight increases the risk for breast cancer recurrence after the primary tumor excision, and significantly reduces the overall survival of patients^13^ by affecting the spreading of breast cancer cells.^14^ This evidence helps to understand the important percentage of women with locally advanced disease upon diagnosis in the Stress Group.

It is well-established that chronic inflammation induced by the excessive availability of lipids found during overweight and obesity conditions can promote cancer spreading and aggressiveness, a process mediated by tumor-driven cytokines.^1^ Together, chronic psychological stress and overweight can impair immunosurveillance as shown in breast cancer patients by Varker et al.^7^ Altogether, these factors are putative stimuli for developing breast tumors with aggressive characteristics.

Based on this hypothesis, we investigated the phenotypic profile of breast tumors in both groups. Our data revealed that women with a history of psychological stress displayed a significant percentage of tumors overexpressing HER2. HER2-amplified breast tumors promote aggressive disease, with poor prognosis^14-16^ due to its rapid proliferation and spreading.^17,18^ These tumors are reported in overweight/obese women, which exhibit enhanced disease spreading in relation to non-obese patients.^8^ In this case, the excessive bioavailability of lipids seems to favor the epithelial transformation of cells into HER2-amplified neoplastic cells_._^19^ Menendez^20^ demonstrated that HER2 cancers take advantage of lipids as their energy source, which is strongly favored in environments rich in fat, such as the breast tissue of overweight or obese women. We further observed that most patients presented with the HER2-amplified tumor mass located in the left breast. The left mammary gland frequently presents more breast tissue than the right, which may benefit fat accumulation and cancer development.^21^

These data support the hypothesis that the women enrolled in the present study have a complex chain formed by historical factors of psychological stress, overweight, and development of phenotypically aggressive breast tumors. Our preliminary findings suggest a “vicious circle” involving chronic psychological stress, overweight, and breast cancer aggressiveness.

The main limitations of our study included the small sample size and the need for a long-term follow-up of patients to examine the recurrence of the disease and responsiveness to chemotherapy.

## CONCLUSION

Our data suggest that chronic psychological stress may represent a considerable risk factor for weight gain and development of aggressive tumors in women diagnosed with breast cancer, such as human epidermal growth factor receptor 2-amplified breast tumors.
