# Supplementary material for: Chronic psychological stress and its impact on the development of aggressive breast cancer
Source: Einstein (Sao Paulo). 2015 Jul-Sep;13(3):352–6. doi: 10.1590/S1679-45082015AO3344 (PMC4943778; doi:10.1590/S1679-45082015AO3344)
Supplement: Translation

# Translation: Estresse psicológico crônico e seu impacto no desenvolvimento de neoplasia mamária agressiva

**Authors:** Thayse Fachin Cormanique, Lirane Elize Defante Ferreto de Almeida, Cynthia Alba Rech, Daniel Rech, Ana Cristina da Silva do Amaral Herrera, Carolina Panis

**Affiliations:** 1 Universidade Estadual do Oeste do Paraná, Francisco Beltrão, PR, Brasil.; 2 Hospital do Câncer de Francisco Beltrão, Francisco Beltrão, PR, Brasil.; 3 Pontifícia Universidade Católica do Paraná, Londrina, PR, Brasil.

**Keywords:** Estresse psicológico, Neoplasias da mama, Sobrepeso, Obesidade, Monitorização imunológica

## Abstract

**Objetivo:**

Investigar os achados clínico-patológicos de mulheres diagnosticadas com câncer de mama e estudar o impacto do estresse psicológico crônico nas características patológicas desses tumores.

**Métodos:**

Investigamos uma coorte composta por mulheres diagnosticadas com câncer de mama divididas em dois grupos. O primeiro foi classificado pela apresentação de estresse psicológico crônico (por meio do *Self-Reporting Questionnaire *− SRQ-20). Outro grupo de mulheres com câncer de mama, mas sem história prévia de estresse psicológico crônico, foi denominado Grupo Controle. Os dados clínicos e patológicos foram avaliados.

**Resultados:**

As mulheres com histórico de estresse crônico apresentaram-se significativamente acima do peso quando comparadas com o Grupo Controle. Além disso, verificou-se que estas mulheres estressadas apresentaram um porcentual significativo de um subtipo de câncer de mama agressivo, o HER2, o que poderia estar associado à possível perda da imunovigilância.

**Conclusão:**

Nossos resultados sugeriram uma ligação entre o estresse psicológico crônico, o excesso de peso e o desenvolvimento de tumores de mama com maior agressividade.

## INTRODUÇÃO

O câncer de mama é uma doença multifatorial, constituindo um problema de saúde pública no mundo todo. Alguns fatores que interagem entre si contribuem para a alta incidência de câncer de mama, como história familiar, presença de genes de alta suscetibilidade, peso corpóreo excessivo e estresse crônico.^1^ ^-^ ^3^

Nesse contexto, o estresse psicológico crônico é um achado comum relatado por pacientes com câncer. Eventos de vida estressantes são considerados importantes componentes que podem afetar o estado emocional dos indivíduos, e sua associação com a perda de apoio social tem relação até com sobrevida significantemente menor em pacientes com câncer de mama.^4^ Há uma associação positiva importante entre sofrimento no início da vida e o desenvolvimento de câncer de mama.^5^ Além disso, uma análise sistêmica de alguns estudos publicados nos últimos 30 anos, que investiga as atribuições causais em pacientes com câncer de mama, demonstrou que sobreviventes de câncer de mama muitas vezes associam sua doença ao sofrimento emocional, além de outros fatores.^6^

Em relação ao impacto biológico do sofrimento crônico, o estresse sustentado por si só pode levar a aumento de peso por meio de vários mecanismos biológicos,^2^ que podem potencialmente resultar em perda da imunovigilância.^7^ As respostas imunes competentes são a maior defesa contra o câncer; desse modo, quando comprometidas, estão fortemente associadas ao desenvolvimento de vários tipos de câncer, incluindo tumores de mama com prognóstico mais reservado.^8^

Apesar de a relação entre desenvolvimento do câncer e estresse crônico ter sido descrita, pouco se sabe a respeito do impacto do estresse psicológico crônico no fenótipo de tumores de mama. Para esclarecer essa questão, investigamos se mulheres que apresentam sofrimento emocional crônico podem exibir fenótipos mais agressivos de câncer de mama.

## OBJETIVO

Investigar os achados clínico-patológicos de mulheres com diagnóstico de câncer de mama, e estudar o impacto do estresse psicológico crônico sobre as características de tumores.

## MÉTODOS

### Desenho do estudo

Este estudo incluiu mulheres residentes no Estado do Paraná, com diagnóstico de carcinoma de mama infiltrativo, durante o período de agosto de 2013 a julho de 2014. Elas tinham sido previamente agendadas para serem submetidas à quimioterapia durante esse mesmo período. A seleção de pacientes foi feita no Centro de Oncologia de Francisco Beltrão (Ceonc), na cidade de Francisco Beltrão (PR) Brasil. Os critérios de inclusão adotados foram mulheres com carcinoma de mama ductal infiltrativo uni- ou bilateral, diagnosticadas entre agosto de 2013 e julho de 2014, elegíveis segundo o *Self-Reporting Questionnaire *(SRQ-20) como estando na coorte de estresse ou sem estress. Para determinar o tamanho da amostra, aplicamos o seguinte cálculo estatístico, em que: N0 = número do tamanho, Z = intervalo de confiança, P = probabilidade, D = margem de erro, n = tamanho da amostra, e N = tamanho da população:



Se levarmos em conta que (1) nossa população tinha aproximadamente 100 mil habitantes; (2) um valor de p de 0,05; (3) segundo a estimativa do Instituto Nacional de Câncer, a incidência de câncer nesta região era de cerca de 61 casos por 100 mil mulheres; e (4) havia cerca de 30 mil mulheres na faixa etária de risco para câncer mamário nessa área, temos que o tamanho de amostra mínimo necessário era de aproximadamente 18 pacientes. Considerando o intervalo do estudo e o fato de que trabalhamos com pacientes previamente agendadas em um dia fixo da semana para se submeterem à quimioterapia, decidimos incluir 17 pacientes em cada grupo. Assim, o estudo incluiu 34 mulheres com diagnóstico de carcinoma de mama ductal infiltrativo. Este estudo foi previamente aprovado pelo Comitê de Ética em Pesquisa, e todas participantes assinaram o Termo de Consentimento Livre e Esclarecido. O projeto de pesquisa foi aprovado pelo Conselho Nacional de Comitê de Ética em Pesquisa Cientifica, número 497.050 e CAAE: 22027213.7.0000.0107. Os prontuários foram avaliados e os dados coletados incluíram idade ao diagnóstico, peso, altura, comorbidades, classificação TNM e esquema de quimioterapia.

Para determinação do estado de estresse psicológico crônico das pacientes, realizamos uma entrevista inicial, para verificar o suporte social da paciente (renda da família, tipo de residência, nível de instrução, estilo de vida e relacionamentos sociais), já que não sabíamos se as pacientes eram de condições sociais diferentes. Já que todas pacientes relataram dados socioeconômicos semelhantes, continuamos o estudo com a aplicação do SRQ-20 para triagem de transtornos psiquiátricos.^9^ Essa entrevista foi aplicada para pacientes que estavam no hospital para tratamento quimioterápico de rotina durante o período do estudo.

As mulheres incluídas neste estudo foram classificadas em dois grupos: Grupo Controle (n=17), formado por mulheres com diagnóstico de câncer de mama sem história pregressa de estresse psicológico crônico; Grupo de Estresse (n=17), formado por mulheres com diagnóstico de câncer de mama com história pregressa de estresse psicológico crônico.

### Subtipagem molecular de tumores de mama por imunoistoquímica

As amostras de biópsias de tumor foram fixadas em formol e incluídas em parafina. Foi realizada imunomarcação com anticorpos primários para o receptor de estrógeno (RE; antirreceptor de estrógeno humano alfa, clone 1D5 a 1:600; Dako, Dinamarca), receptor de progesterona (RP; antirreceptor de progesterona humana, clone PGR 636 a 1:500; Dako, Dinamarca), e receptor tipo 2 do fator de crescimento epidérmico humano (HER-2, anti-HER2-pY-1248 humano, clone PN2A a 1:500; Dako, Dinamarca), junto de um *kit* comercial de imunoistoquímica. As amostras foram consideradas positivas para RE/RP quando pelo menos 10% dos núcleos das células tumorais estavam corados. A superexpressão de HER2 foi detectada por coloração forte da membrana (3+), ou quando a amplificação de HER2, em amostras com coloração moderada (2+) de membrana, foi observada em análises por *Fluorescent in Situ Hybridization* (FISH). As amostras foram pontuadas e classificadas, sendo consideradas HER2-positivas quando o escore HER2 IHC foi 3+, e HER-negativas, quando o escore foi 1+ ou zero. As amostras com escore 2+ foram analisadas por FISH para detectar amplificação de HER2 (HER2 FISH pharmDx^TM^; Dako, Dinamarca). As amostras com escore IHC 2+ e resultado amplificado em FISH foram consideradas HER2-positivas, enquanto amostras com um escore IHC 2+ e sem amplificação em FISH foram HER2-negativas.^10^

### Análise dos dados

Para parâmetros clínico-patológicos, os dados foram expressos como média ± erro padrão da média. Todos os dados foram comparados usando o teste não paramétrico de Mann-Whitney. Valor de p<0,05 foi considerado significativo. Todas as análises estatísticas foram feitas usando o programa GraphPad Prism versão 5.0 (GraphPad Prism Software, San Diego, Califórnia, Estados Unidos).

## RESULTADOS

A média de idade ao diagnóstico foi de 60,2 anos. Observamos que 40% das pacientes tiveram diagnóstico de câncer de mama por mamografias de rotina, e 53% relataram a percepção de nódulos nas mamas por autoexame. Os outros 7% não tinham nódulos palpáveis, mas sentiam dor na mama e procuraram um médico por esse motivo. Com relação à verificação de estresse psicológico crônico (Tabela 1), observou-se que 79% relataram um histórico de estresse psicológico crônico. Os traumas mais comuns relatados foram morte de membros da família, abandono por um parceiro, perda de emprego, abuso sexual e depressão maior. Além disso, 73% das pacientes associaram a ocorrência de estresse psicológico crônico com o desenvolvimento de câncer de mama. No total, 47% foram classificadas como potenciais portadoras de distúrbios psíquicos pelo SRQ-20. As pacientes que relataram história positiva de estresse psicológico foram classificadas para formar o Grupo de Estresse.

**Tabela 1:** Principais parâmetros empregados para caracterização do estresse psicológico crônico

Parâmetro	Porcentagem média
Relações familiares	
Boas	85
Tipos de sofrimento psicológico relatados	
Morte de parentes	36
Perda de emprego	9
Abandono por parceiro	18
Abuso sexual	9
Depressão	9
Outro sofrimento emocional	19
Recebeu apoio durante o tratamento	
Sim	93
Apresentou dificuldade em abandonar o trabalho	
Sim	57
Acredita que o estresse crônico está associado como causa da doença	
Sim	73
Risco de desenvolver transtornos mentais	
Sim	47

Com o objetivo de compreender o impacto clínico-patológico do estresse psicológico crônico sobre o câncer de mama e seus aspectos clínicos, comparamos os dados do Grupo de Estresse com uma coorte controle, composta por mulheres diagnosticadas com câncer de mama sem história pregressa de estresse emocional crônico. A figura 1 mostra que o Grupo Controle foi composto principalmente por mulheres com câncer de mama que apresentavam um índice de massa corporal (IMC) normal (90%). Por outro lado, as pacientes do Grupo de Estresse apresentaram predominância significativa de mulheres com sobrepeso (54%; p=0,0041). A subtipagem molecular de tumores de mama (Figura 2) indicou a predominância de tumores HER2 positivos no Grupo de Estresse em comparação com os controles (31±1,41% *versus* 12±0,05%; p=0,0136).

**Figura 1:** Análise de índices de massa corporal. Usamos a Classificação Internacional de Índice de Massa Corporal para classificar as pacientes em peso normal (IMC até 25kg/m2) ou sobrepeso (IMC acima de 25kg/m2)

**Figura 2:** Subtipagem molecular de tumores. As imagens representam a marcação de IHC para HER2

## DISCUSSÃO

Nos últimos anos, o câncer de mama tem sido a principal neoplasia maligna em mulheres de todo o mundo. Esse fato merece atenção, pois a lista de fatores de risco relacionados com essa doença está crescendo. Nosso objetivo foi investigar mulheres com diagnóstico de câncer de mama que relatavam história pregressa de estresse psicológico crônico e seu impacto sobre os aspectos clínico-patológicos do câncer de mama. Para alcançar esse objetivo, primeiro aplicamos o SRQ-20 como uma ferramenta de triagem psiquiátrica, para compor a coorte de estresse.

O SRQ-20 é uma ferramenta desenvolvida pela Organização Mundial da Saúde (OMS) para rastrear transtornos psiquiátricos em pacientes. O questionário detecta sintomas como ansiedade, depressão e queixas psicossomáticas com acurácia adequada.^11^ O SRQ-20 já foi aplicado anteriormente e validado para a população brasileira,^9^ incluindo pacientes com câncer de mama.^12^ Nossos dados demonstram que cerca de 47% das pacientes atendidas durante o período de aplicação do SRQ-20 foram classificadas como portadoras de sofrimento psiquiátrico e incluídas no Grupo de Estresse.

Depois, selecionamos outra coorte de pacientes com uma história semelhante (idade ao diagnóstico, história familiar, estado geral de saúde e renda familiar), mas sem relato anterior de estresse psicológico crônico para compor o Grupo Controle. Empregamos esta abordagem com o propósito de comparar o impacto de sofrimento emocional crônico sobre aspectos clínicos e patológicos de tumores mamários, em ambos os grupos. Nossos dados indicam que uma porção significativa das mulheres incluídas no grupo de Estresse apresentava excesso de peso em comparação com o Grupo Controle. Tanto o estresse psicológico como o excesso de peso são importantes fatores de risco para o desenvolvimento de câncer de mama.^1^ ^,^ ^2^ Em condições de estresse crônico, há um aumento sistêmico de níveis de cortisol, o que aumenta o apetite e favorece o armazenamento de lipídios em tecido adiposo.^2^ Este conjunto de eventos biológicos, que contribuem para o ganho de peso, é composto por fatores de risco relevantes para câncer de mama na sociedade moderna,^8^ e pode ajudar parcialmente a compreender os resultados encontrados no presente estudo. O sobrepeso aumenta o risco de recorrência de câncer de mama após a remoção do tumor primário e reduz de forma significativa a sobrevida global de pacientes,^13^ ao interferir na disseminação de células de câncer de mama.^14^ Essas evidências ajudam a entender a importante porcentagem de mulheres com doença avançada localmente ao diagnóstico no Grupo de Estresse.

Já foi bem estabelecido que a inflamação crônica induzida pela disponibilidade excessiva de lipídios encontrados em casos de sobrepeso e obesidade pode promover a disseminação e a agressividade do câncer, um processo mediado por citocinas dirigidas pelo tumor.^1^ Juntos, o estresse psicológico crônico e o sobrepeso podem prejudicar a imunovigilância, como demonstrado por Varker et al.^7^ em pacientes com câncer. No total, esses fatores são supostos estímulos para o desenvolvimento de tumores mamários com características agressivas.

Com base nessa hipótese, investigamos o perfil fenotípico de câncer de mama em ambos os grupos. Nossos dados revelaram que mulheres com histórico de estresse psicológico demonstraram porcentagem significativa de tumores com superexpressão de HER2. Os tumores de mama com amplificação de HER2 causam doença agressiva, com pior prognóstico^14^ ^-^ ^16^ devido à rápida proliferação e à disseminação.^17^ ^,^ ^18^ Esses tumores são descritos em mulheres com sobrepeso/obesas, que exibem maior disseminação da doença em relação a pacientes não obesas.^8^ Nesse caso, a biodisponibilidade de lipídios parece favorecer a transformação epitelial de células em células neoplásicas com HER2 amplificado.^19^ Menendez^20^ mostrou que cânceres HER2 aproveitam os lipídios como sua fonte de energia, o que é fortemente favorecido em ambientes ricos em gordura, como o tecido mamário de mulheres com excesso de peso ou obesas. Também observamos que a maioria das pacientes apresentou a massa tumoral de HER2 amplificada na mama esquerda. A glândula mamária esquerda frequentemente apresenta mais tecido mamário que a direita, o que pode beneficiar o acúmulo de gordura e o desenvolvimento de câncer.^21^

Estes dados fortalecem a hipótese de que as mulheres incluídas no presente estudo tenham uma cadeia complexa formada por fatores históricos de estresse psicológico, sobrepeso e desenvolvimento de tumores mamários fenotipicamente agressivos. Nossos achados preliminares sugerem um “ciclo vicioso”, envolvendo estresse psicológico crônico, sobrepeso e agressividade do câncer mamário.

As principais limitações do nosso estudo incluíram o tamanho pequeno da amostra, e a necessidade de um seguimento de longo prazo de pacientes, para examinar a recorrência da doença e a resposta à quimioterapia.

## CONCLUSÃO

Nossos dados sugeriram que o estresse psicológico crônico pode representar um considerável fator de risco para ganho de peso e desenvolvimento de tumores agressivos em mulheres com diagnóstico de câncer de mama, como tumores mamários com amplificação do receptor do fator de crescimento epidérmico humano 2.
